# Research on Lane Line Detection Algorithm Based on Instance Segmentation

**DOI:** 10.3390/s23020789

**Published:** 2023-01-10

**Authors:** Wangfeng Cheng, Xuanyao Wang, Bangguo Mao

**Affiliations:** 1 School of Mechanical Engineering, Anhui University of Science and Technology, Huainan 232001, China; 2 Institute of Environment-Friendly Materials and Occupational Health, Anhui University of Science and Technology, Wuhu 241000, China; 3 Shaanxi Automobile Holding Group Huainan Special Purpose Vehicle Co., Ltd., Huainan 232001, China

**Keywords:** autonomous driving, complex road environment, lane line detection, instance segmentation

## Abstract

Aiming at the current lane line detection algorithm in complex traffic scenes, such as lane lines being blocked by shadows, blurred roads, and road sparseness, which lead to low lane line detection accuracy and poor real-time detection speed, this paper proposes a lane line detection algorithm based on instance segmentation. Firstly, the improved lightweight network RepVgg-A0 is used to encode road images, which expands the receptive field of the network; secondly, a multi-size asymmetric shuffling convolution model is proposed for the characteristics of sparse and slender lane lines, which enhances the ability to extract lane line features; an adaptive upsampling model is further proposed as a decoder, which upsamples the feature map to the original resolution for pixel-level classification and detection, and adds the lane line prediction branch to output the confidence of the lane line; and finally, the instance segmentation-based lane line detection algorithm is successfully deployed on the embedded platform Jetson Nano, and half-precision acceleration is performed using NVDIA’s TensorRT framework. The experimental results show that the Acc value of the lane line detection algorithm based on instance segmentation is 96.7%, and the FPS is 77.5 fps/s. The detection speed deployed on the embedded platform Jetson Nano reaches 27 fps/s.

## 1. Introduction

Lane line detection is a crucial component of the road surface information and environment perception used in autonomous driving technology, which contains semantic information about road areas, identifies the direction of travel, and enhances guidance data. Because deep learning and artificial intelligence have advanced so quickly, lane line detection technology is now able to provide automated driving vehicles with collision warning, lane departure warning, and auxiliary environment perception information, as well as to help the system realize lane path planning [1,2]. This increases the safety of automated driving.

Lane lines, the most important road traffic signs, constrain vehicles’ driving paths. Traditional lane detection algorithms based on features and models and deep learning-based lane line detection algorithms are the two broad categories of current mainstream lane line detection algorithms.

The traditional lane line detection algorithms first needed to preprocess the image according to the specific use scene to eliminate noise interference; then, used the preset shape, color, or spatial features to match the road image for feature extraction; and finally, used the least-squares algorithm to simulate the lane line. Mammeri et al. [3] proposed a lane line detection system combining the most stable extremal region and Hough transform, which used matching features such as the color and shape of lane lines, to detect lane lines. Sotelo et al. [4] developed a road segmentation algorithm based on an HIS color space and a two-dimensional constrained space for obtaining the lane line information. Ozgunalp et al. [5] proposed a feature-map-based lane detection algorithm that used an inverse perspective transformation method. Chi et al. [6] proposed the use of the road vanishing point estimation algorithm to detect lane lines, but the model-based algorithm was computationally complex. In addition, the model-based detection algorithm was computationally intensive and could only deal with problems such as road occlusion in specific environments, thus making the results somewhat limited. Traditional lane line detection algorithms based on features and models [7,8,9,10,11] were easily affected by external environmental factors. When the lane line was broken, blocked, or unpainted, its robustness was extremely low, which would lead to incorrect lane line detection or even make it impossible. To solve the problem of low accuracy of lane line detection in complex road environments, convolutional neural networks based on deep learning were widely used in lane line detection because of their powerful feature detection capabilities. Aly et al. [12] used Gaussian filtering and detected street lanes using line detection and a new RANSAC spline-fitting technique. Kim et al. [13] combined convolutional neural networks with the RANSAC algorithm and proposed a continuous end-to-end migration learning method that can detect both left and right lane lines of the current lane. Neven et al. [14] transformed the lane line detection problem into an instance segmentation problem that distinguished the lane lines and their background using a binary classification principle. Ren et al. [15] proposed a Fast-RCNN network, which used a multi-task loss function for training, which allowed all layers to be updated and reduced the number of parameters in fully connected layers, improving detection performance. However, the two-stage-based network detection was slow. He et al. [16] proposed the use of SPP-Net to improve the detection speed. By introducing the pooling layer to reduce the parameter amount of the model, the speed of the model was improved to a certain extent, but the detection accuracy decreased to a certain extent. Hairs et al. [17] proposed an ak-cnn model for lane line detection, which had an auxiliary loss, which reduced parameters and running time while improving detection and estimation indicators and had an excellent real-time performance. However, it was prone to a lack of flexibility in complex traffic conditions. Liu et al. [18] proposed a transformer-based network structure to better learn lane structure information and context information and directly output the parameter information of lane lines to avoid additional post-processing, and improved the overall detection speed. However, due to the large number of candidate lane lines generated in the network, post-processing methods (NMS) were still required to filter redundant lane lines, and the real-time performance was poor. Chao et al. [19] proposed a VGG-ss model to build an encoder–decoder structure to improve the accuracy and real-time performance of lane line detection. However, when the lane line was blocked or destroyed, the precision measurement accuracy and real-time speed dropped slightly, and this experiment was only used for pictures and had not been studied on videos, sports, and other images.

In summarizing the aforementioned literature, it can be seen that the current lane line detection algorithms based on deep learning use data to extract features adaptively, which makes the lane line detection accuracy and real-time detection speed significantly improved compared with traditional lane line detection algorithms. However, under factors such as road occlusion, road blur, and the characteristics of slender and sparse lane lines, it is difficult for ordinary convolutional neural networks to extract accurate road features from road images, and cannot achieve satisfactory accuracy and real-time performance. Aiming at the above problems, this paper proposes a lane line detection algorithm based on instance segmentation. The contributions part of this paper includes the succeeding three points:

1. Improve the RepVgg-A0 network to expand the receptive field of the network without increasing the amount of calculations, and propose a multi-size asymmetric shuffled convolution model to enhance the extraction of sparse and slender lane lines ability.
2. An adaptive upsampling model is proposed, which allows the network to select the weight of the two upsampling methods at each position; at the same time, a lane line prediction branch is added to facilitate the output of lane line confidence.
3. Deploy the lane line detection algorithm to the embedded platform Jetson Nano, and use the TensorRT framework for half-precision acceleration to make its detection speed meet the needs of real-time detection.

This paper is arranged as follows: A lane line detection model based on instance segmentation is designed in Section 2. In Section 3, the lane line detection experiment is carried out by combining the Tusimple extended dataset and the video collected by the real car and deployed to the mobile terminal. Finally, in Section 4, the content of this paper is summarized and directions for future work are provided.

## 2. Design of Lane Line Detection Model

Refer to the encoder–decoder network structure [19] to build a lane line detection instance segmentation model. The lane line detection model framework based on instance segmentation includes an encoder, a feature enhancement model, a decoder, and a lane prediction branch. Firstly, the encoder part uses the improved lightweight network RepVgg-A0 to encode the road image; secondly, the feature enhancement model uses a multi-scale asymmetric shuffled convolution model to enhance the ability to extract lane line features; further, the adaptive upsampling model is used as the decoder, the feature map is upsampled to the original resolution for pixel-level classification and detection, and the lane line prediction branch is added to output the lane line confidence.

### 2.1. Design of Encoder Network Structure

After the road image is input, the lightweight network RepVgg-A0 is used as the encoder of the model to initially extract the features of the lane line. RepVgg follows the lightweight model design guidelines proposed in ShuffleNet V2 [20] and proposes the idea of structural reparameterization. Different model structures are used in the training and inference stages, and different branch structures are cleverly fused during inference to reduce memory usage and speed up model inference. This is also one of the reasons why the lane line detection algorithm in this paper has a faster inference speed.

The lightweight RepVgg-A0 [21] network downsamples and compresses the input image to 1/32 of the original image through 3 convolutional layers with a step size of 2, reducing the image resolution while increasing the receptive field. However, too small a resolution will, as a result, cause the encoded image to lose a lot of spatial information, and it is difficult for the subsequent decoding process to repair this information, which affects the accuracy of lane line detection. Therefore, the RepVgg-A0 network structure is adjusted, and the step size of the convolution in the last 2 layers of the network is set to 1 so that the downsampling ratio is reduced from 32 times to 8 times. After the above operations, the size of the encoded feature map is relatively increased, and more original information about the lane line is retained, but another problem arises. The receptive field of the network becomes smaller, and it is difficult for it to learn global features. Therefore, the hole convolution is introduced in the last 2 layers of the network, and the conventional 3 × 3 convolutions in the last 2 layers of the RepVgg-A0 network are replaced by 3 × 3-hole convolutions with a hole rate of 2 and 4, without introducing additional calculations.

**Figure 1:**
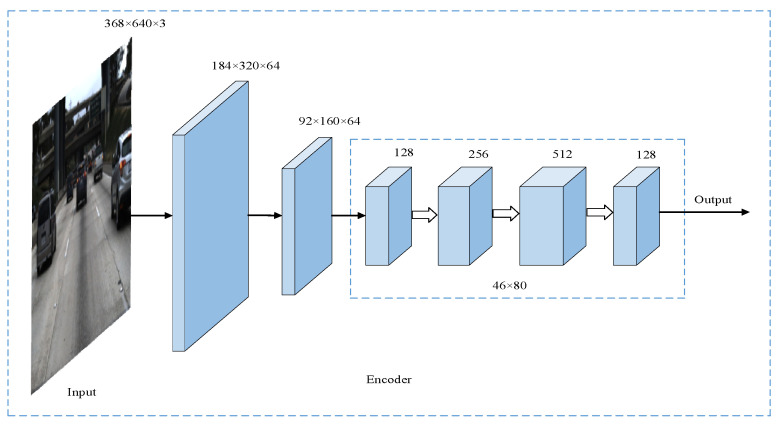
Improved encoder network structure diagram.

After the input 3-channel image is initially extracted by the improved RepVgg-A0 network, the number of channels becomes 1280, and the resolution is reduced to 1/8 of the original. To reduce the calculation amount of subsequent operations and fuse the extracted features at the same time, 1 × 1’s convolutions are added to the last layer of the encoder to compress the number of channels to 128. After the above steps, the overall structure of the encoder is shown in Table 1, and its network structure is shown in Figure 1 below.

### 2.2. Design of Feature Enhancement Model

**Figure 2:**
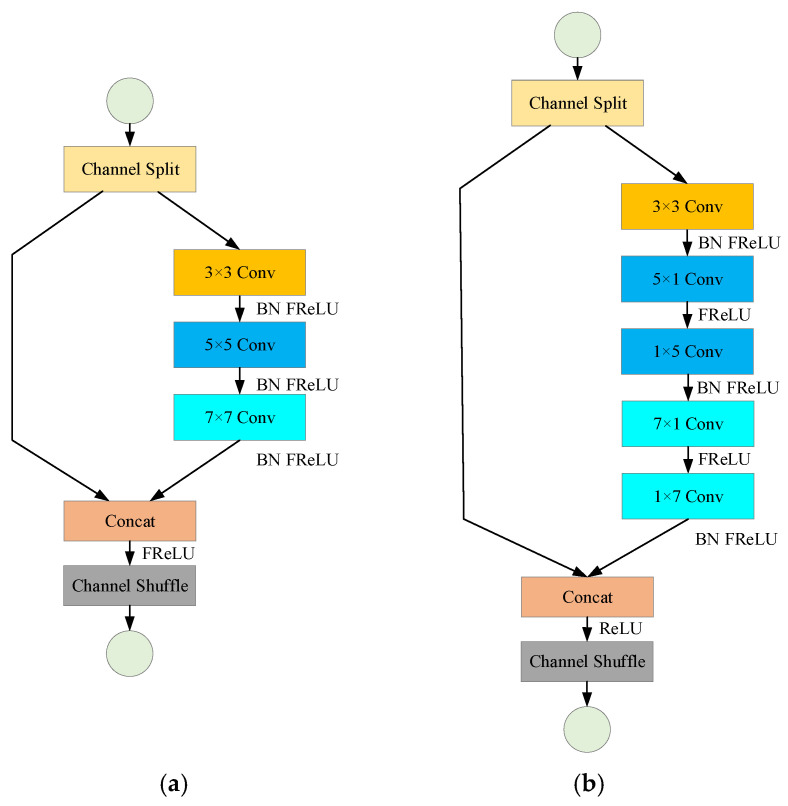
Multi-scale shuffled convolution module and multi-scale asymmetric shuffled convolution module. (**a**) Multi-scale shuffled convolution module. (**b**) Multi-scale asymmetric shuffled convolution module.

Lane line detection is different from conventional object detection. A lane line usually spans the entire image, which requires the network to have a large enough receptive field. For the instance segmentation network, an effective way to increase the receptive field is to use a larger convolution kernel. Inspired by the ShuffleNet V2 network, this paper designs a multi-size shuffled convolution module containing 3 sizes of convolution kernels of 3 × 3, 5 × 5, and 7 × 7. Among them, 3 × 3 convolutions are used to extract the detailed features of lane lines, and 5 × 5 and 7 × 7 convolutions have larger receptive fields, which can capture larger-scale lane line features. The multi-size shuffling convolution module structure is shown in Figure 2a. After the feature map is input, firstly, the channel is divided into two branches, and the secondary branch performs the same mapping. Secondly, the main branch performs convolutions of three sizes of 3 × 3, 5 × 5, and 7 × 7 in sequence, and uses the FReLu activation function [22] to add nonlinear factors after each convolution. Finally, after splicing the main branch and the second branch channels, the full channel shuffling operation is performed to promote the fusion of feature information between channels.

The computational cost of using shuffled convolution modules is somewhat lower than using large convolution kernels directly, but the 5 × 5 and 7 × 7 convolutions still require a lot of calculations. In order to further simplify calculations, asymmetric convolutions are introduced in this paper. Asymmetric convolution reduces the amount of calculation by substituting *k* × 1 and 1 × *k* convolutions for traditional *k* × *k* convolutions. The convolution calculations and parameters for the standard *k* × *k* convolutions are as follows:

(1) $$Params=k^{2}C_{i}C_{o}$$

(2) $$FLOPs=k^{2}C_{i}C_{o}H_{o}W_{o}$$

Among them are the height and width of the input feature map, respectively, and the channel numbers of the input and output feature maps, respectively. The asymmetric convolution parameters and calculations equivalent to *k* × *k* convolution are as follows:

(3) $$Params_{a}=2kC_{i}C_{o}$$

(4) $$FLOPs_{a}=kC_{i}C_{o}H_{o}(2W_{o}+k-1)$$

It can be seen from Formulas (1)–(4) that the larger the size of the convolution kernel, the more obvious the number of parameters and calculations that can be reduced by converting it into an asymmetric convolution. In addition, some studies have shown that the asymmetric effect of convolution applied to the middle layer of the network is better [23]. Therefore, the 5 × 5 and 7 × 7 convolutions in the multi-size shuffled convolution module are replaced by asymmetric convolution, and a multi-size asymmetric shuffled convolution module, as shown in Figure 2b, is designed. For fixed 46 × 80 × 128 input feature maps, the number of module parameters and calculations are reduced by 60.24% and 61.47%, respectively.

**Figure 3:**
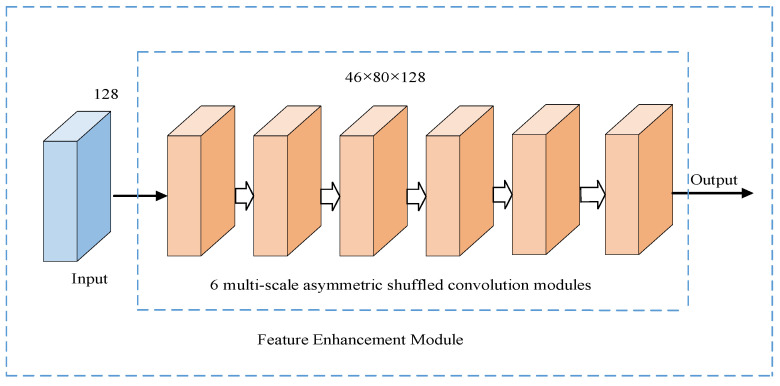
Feature enhancement module.

Stack 6 multi-size asymmetric shuffled convolution models are used to form the feature enhancement model of the lane line detection model in this paper. Among them, the last 5 modules use hole convolution to further expand the receptive field, and the hole rates are set to 2, 4, 6, 8, and 10, respectively. The feature enhancement module further extracts the lane line information existing in the feature map output by the encoder and inputs the result into the lane line prediction branch and decoder structure, and its corresponding network structure is shown in Figure 3 below.

### 2.3. Design of Decoder Network Structure

The decoder’s job is to classify each pixel in the feature map by upsampling the low-resolution feature map, which contains rich feature information, to the size of the input image. The two most popular upsampling algorithms are bilinear interpolation and transposition convolution. However, these algorithms ignore the impact of the gradient in pixel values between adjacent points, which will degrade the sampled image’s detailed features. It is also simple to ignore coarse-grained features and other issues. To solve the above problems, Zheng et al. [24] proposed a bilateral upsampling module, which directly adds bilinear interpolation and transposed convolution upsampling results, and achieved certain results, but did not consider the two kinds of upsampling based on the applicability of the method to a specific image area. To effectively extract image features, this paper suggests an adaptive upsampling module that enables the network to choose the weight of the two upsampling methods at each location.

**Figure 4:**
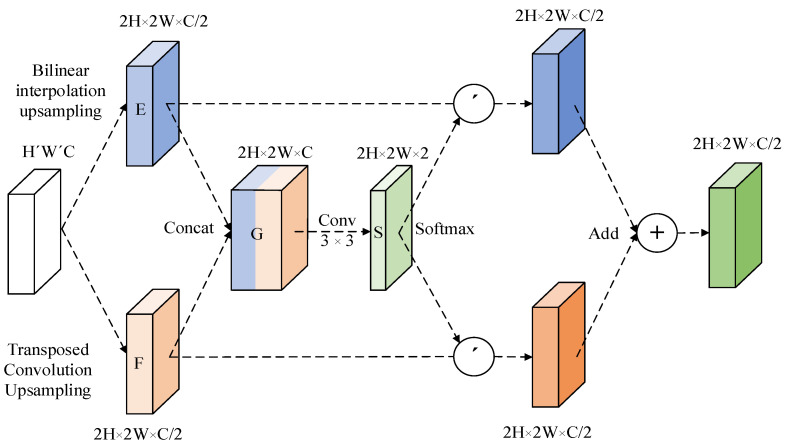
Adaptive upsampling module.

The adaptive upsampling module structure is shown in Figure 4. After inputting the H × W × C feature map, firstly, use bilinear interpolation and transposed convolution to perform upsampling, and initially obtain two 2H × 2W × C/2 upsampling feature maps, E and F; then, splice E and F at the channel dimension to obtain a 2H × 2W × C feature map G, perform 3 × 3 convolutions on G to extract the spatial attention description S (2H × 2W × 2), and use the Softmax function to extract two attention weights of 2H × 2W × 1 for S; finally, the attention weights are weighted and summed with E and F, respectively, to obtain the final upsampling result (2H × 2W × C/2).

**Figure 5:**
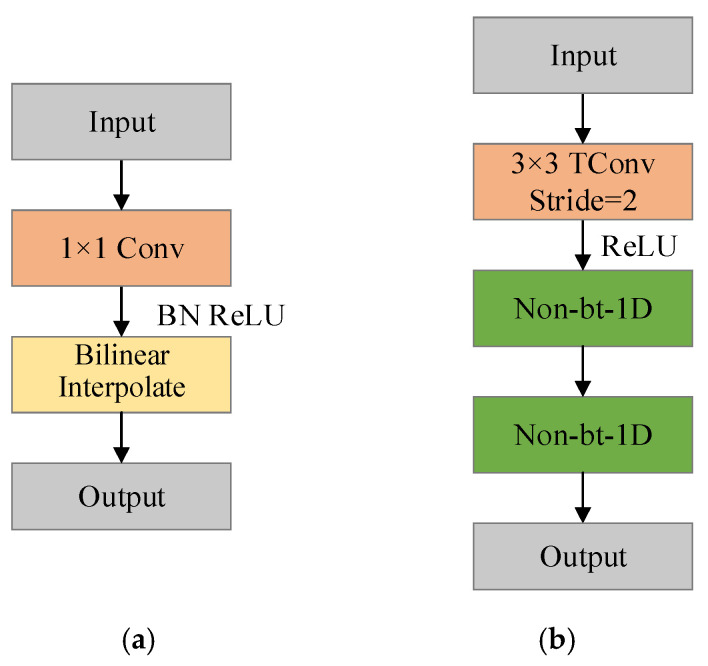
The specific structure of the two upsampling modules. (**a**) Bilinear interpolation upsampling. (**b**) Transposed convolution upsampling.

The bilinear interpolation upsampling structure in the adaptive upsampling module is shown in Figure 5a. Firstly, 1 × 1 convolutions are used to reduce the number of channels to a half, and then bilinear interpolation is performed to double the size of the feature map. The upsampling structure of the transposed convolution is shown in Figure 5b. The transposed convolution with a step size of 2 is used to expand the size of the feature map by 2 times while compressing the number of channels, and then accessing two asymmetric convolutions-Non-bt-1D modules [25].

**Figure 6:**
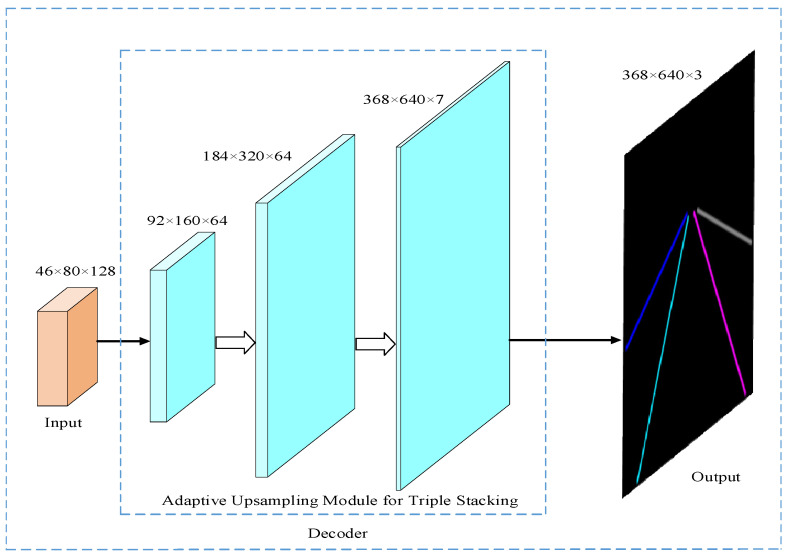
Decoder network structure.

The adaptive upsampling module is superimposed three times to form the decoder of the lane line detection model in this paper, and its corresponding network structure is shown in Figure 6. The input feature map is decoded by the decoder and upsampled to the original image size, and the number of channels is reduced to 7. The first channel is used to predict the background of the lane line, and the other channels directly predict the pixel coordinates of the lane line instance, which has a faster detection speed than the algorithm that is first semantically segmented and then fits the lane line.

### 2.4. Design of Lane Line Prediction Branch

**Figure 7:**
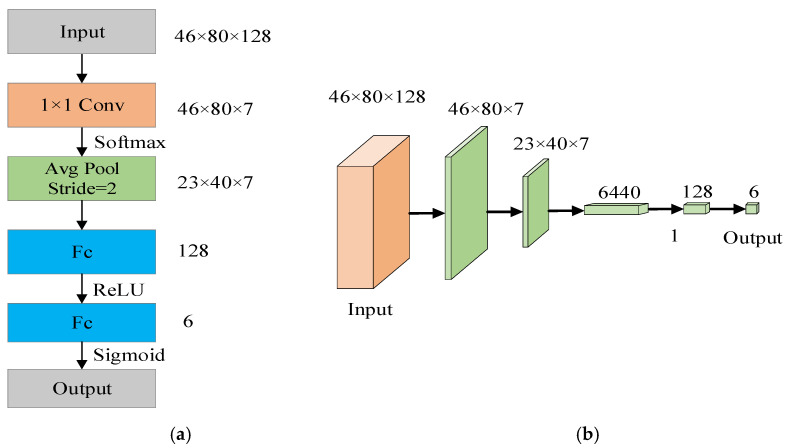
Lane line prediction branch. (**a**) Internal structure of lane line prediction branch. (**b**) External structure of lane line prediction branch.

This paper develops a lane line prediction branch to assess the existence of each lane line and determine the degree of confidence in the existence of output lane lines. Figure 7a depicts the network’s internal structure, and Figure 7b depicts the network’s external structure. Firstly, the number of channels is reduced to 7 through 1 × 1 convolution, and after activation by Softmax, average pooling with a step size of 2 is used to downsample to 23 × 40 × 7; then, two fully connected layers are used continuously and activated by ReLU and Sigmoid, respectively, and output length is a one-dimensional feature vector of 6, respectively, representing the probability of the existence of 6 pre-selected lane lines. In actual use, set a confidence threshold. When the confidence is greater than the threshold, it means that the lane line exists, otherwise it does not exist. This paper sets the threshold to 0.5.

### 2.5. Proposed Lane Detection Model

**Figure 8:**
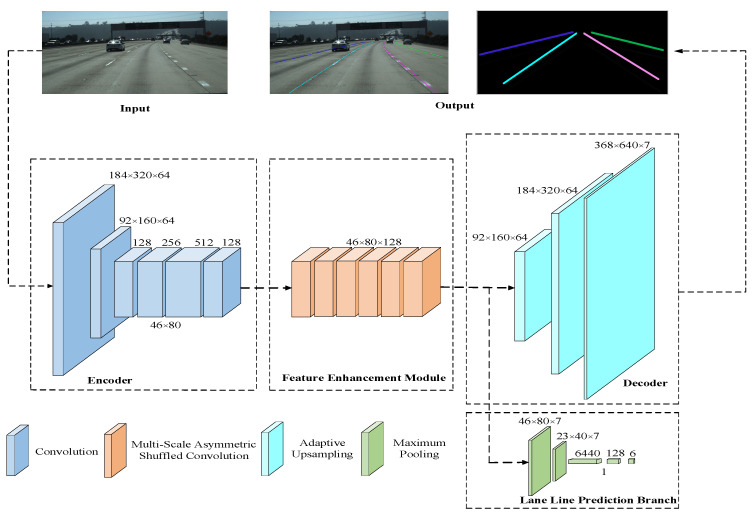
Lane detection model.

Figure 8 depicts the lane line detection model based on instance segmentation. This model uses ReLU as the activation function and primarily consists of 12 convolutional layers, 3 upsampling layers, and 5 pooling layers. The input feature map is compressed three times by the encoder, and the output feature map is stacked by six multi-size asymmetric shuffled convolutions through the feature enhancement model. Part of the output feature map is passed to the decoder, and the other part is passed to the lane line prediction branch. The output feature map is adaptively up-sampled 3 times by the decoder, and the result of the instance segmentation of the feature map is output. The lane line prediction branch outputs the lane line confidence through maximum pooling 5 times. The encoder network structure and the decoder network structure present an asymmetric state, which effectively reduces the number of parameters and computation of the model.

## 3. Experimental Results and Analysis

### 3.1. Dataset and Preprocessing

**Figure 9:**
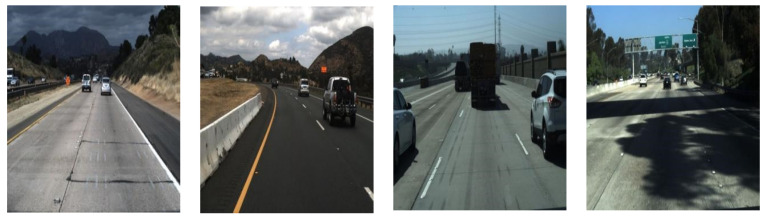
Display of some scenes from the TuSimple dataset.

This paper is based on the TuSimple [26] dataset, which comprises video images collected on American highways. There are 20 frames in each segment. The original dataset only marked the final frame of the 20 frames because there are many video frame data points. The first frame and the images of the tenth and eleventh frames in the middle are chosen for labeling to improve the dataset’s generalizability. The labeling file is in json format, and for every ten pixels in the expanded (vertical) direction, a point is marked. There are 25,632 pictures of roads. Different from the original dataset, 14,504 pictures are selected for training, 2325 pictures are used for verification, and 8803 pictures are used for testing. To enhance the diversity of the data and improve the robust effect of the model, data enhancement processing is performed on the training set, including random rotation and random horizontal deflection. Figure 9 shows some common scenes in the dataset. Each image has 2 to 5 marked lane lines. In this paper, these discrete lane line coordinate points are connected to form an example image as a real mark.

### 3.2. Experiment Preparation

The server used in the experiment is the 11th Gen Intel (R) Core (TM) i5-11400H @ 2.70 GHz 2.69 GHz, 512 GB memory, and NVIDIA GeForce RTX3050 graphics processor. The operating system is Windows 10 professional version, the deep learning framework is tensorflow2.4-GPU, and CUDA version is 11.0.

**Figure 10:**
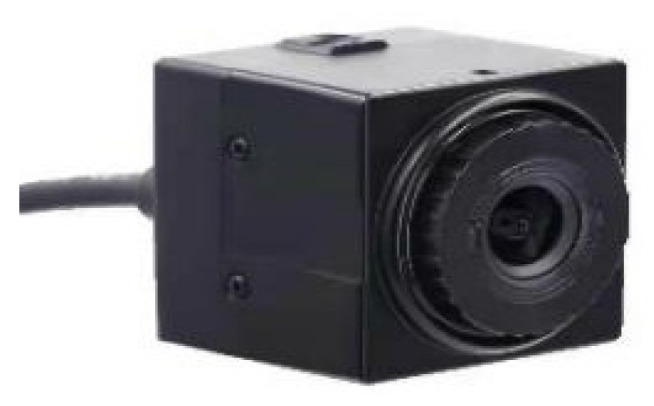
Image acquisition equipment.

**Figure 11:**
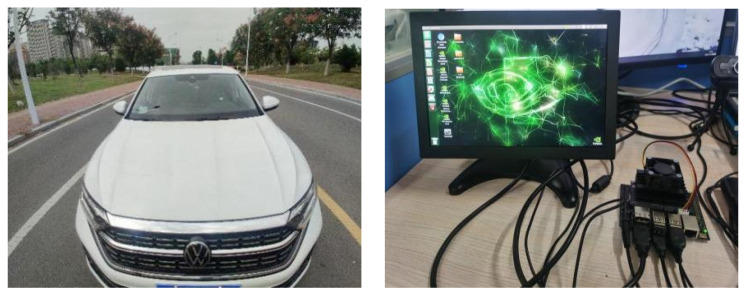
Experimental vehicle and Jetson Nano embedded platform.

The video acquisition device used in the experiment is a front-view camera, as shown in Figure 10, with a resolution of 2592 × 1944. The experimental vehicle is a Volkswagen Sagitar, the embedded platform Jetson Nano is used for mobile deployment, and the operating system is ubuntu18.0.4. The details are shown in Figure 11.

Table 2 displays the lane line detection model’s hyperparameter settings. For lane line image segmentation and lane line confidence prediction, different loss functions—the cross-entropy loss function and binary cross-entropy loss function, respectively—are used. Use each batch for training after updating the model parameters, and record it as a training session. Set the maximum number of iterations to 300, and the maximum number of training times to 80,000. When the number of training times is greater than this value, stop training. The learning rate is determined by the following formula:

(5) $$L={\left(1-\frac{s}{p}\right)}^{0.9}$$

In the formula, *L* represents the learning rate, is the current training times, and is the highest training times.

### 3.3. Model Evaluation Index and Performance Comparison of Different Models

The performance evaluation of the lane line detection model in this paper is performed using the official evaluation method provided by TuSimple. Each detected lane line is represented by a set of x-axis coordinates with a fixed y-axis. The difference between the number of detected lane lines and the number of real lane lines cannot be greater than two, otherwise, it is judged that no lane line is detected. The evaluation indicators include accuracy rate (Acc), false positive rate (*FP*), false negative rate (*FN*), parameter amount (Params), floating point calculation amount (FLOPs), and running speed (FPS). Accuracy is calculated as follows:

(6) $$\left\{ \begin{array}{l} AP=\int_{0}^{1}PRdr\\ mAP=\frac{1}{C}\sum_{Ci\in C}AP_{\left(Ci\right)} \end{array}\right.$$

wherein $N_{pred}$ is the number of correctly detected lane line points and $N_{gt}$ is the number of real lane line points. The false positive rate and false negative rate are calculated as follows:

(7) $$FP=\frac{F_{pred}}{N_{pred}}$$

(8) $$FN=\frac{M_{pred}}{N_{\mathrm{gt}}}$$

Among them, $F_{pred}$ is the number of wrongly predicted lane lines, $M_{pred}$ means the number of real lane lines that have not been predicted, and the lower the values of *FP* and *FN*, the better the model performance.

To verify the performance of the model in this paper, it is compared with existing models (ResNet-18, ResNet-34 [27], Enet [28], LaneNet [29], SCNN [30], ENet-SAD [31], RESA-50 [32], SGLD-34 [33], Res34-VP [34]) that conducted comparative experiments on the TuSimple test set, and the results are shown in Table 3.

As can be seen from Table 3, the lane detection model proposed in this paper is superior to the current excellent lane detection model in terms of accuracy, achieving the highest accuracy rate and the lowest FP value. Moreover, the amount of parameters and calculations of the model is only higher than that of the lightweight network Enet, and the reasoning speed is second only to Res18-Seg. The model in this paper can quickly and accurately detect lane lines with a small number of computing resources, achieve a balance between accuracy and speed, and meet the accuracy and real-time requirements of lane line detection. Therefore, on the whole, the lane detection model in this paper is superior to other lane detection models in terms of comprehensive performance.

To verify the influence of the adaptive upsampling module and the feature enhancement module on the overall performance of the lane line detection model, an ablation experiment was carried out to compare the accuracy before and after adding the adaptive upsampling module and the feature enhancement module. Table 4 records the results of the ablation experiment.

It can be seen from Table 4 that after adding the adaptive upsampling module and the feature enhancement module, the accuracy of the model has been improved to varying degrees, which are 0.2% and 0.82%, respectively. After using the two modules comprehensively, the accuracy rate of the model is increased to 96.7%, which is 0.89% higher than the original, indicating that the above two modules can effectively improve the performance of the lane line detection model.

### 3.4. Comparison of Loss Function Curves

**Figure 12:**
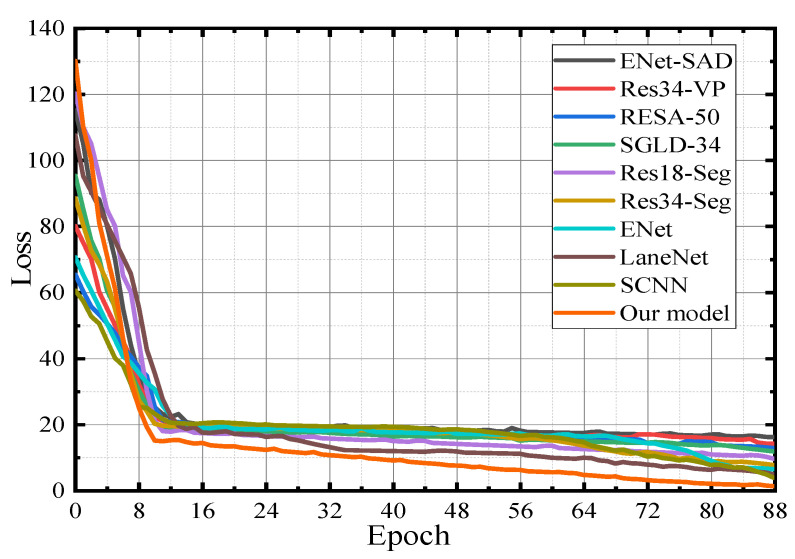
Model verification—loss function graph.

In lane line detection, the rationality, quality, and performance of the first nine lane line detection algorithms in Table 3 are tested to compare them with the model in this paper. Figure 12 is the verification curve of the loss value during the model training process. The maximum number of iterations set in the experimental environment is 300, and the maximum number of training is 80,000. The number of iterations is converted to 89 generations, that is, it is executed from 0 to 88 generations. The initial learning rate of the SGD optimizer is 0.02. When the above nine lane line detection algorithms converge to a specific stage, the convergence speed of the loss function decreases significantly due to the decline in the feature extraction ability of the model. After the convergence speed decreases, the loss error curves of the first nine lane line detection algorithms in Table 3 oscillate greatly during the training process. From the ENet-SAD, Res34-VP, RESA-50, SGLD-34, and Res18-Seg loss function curves, it can be seen that the loss function converges faster in the early stage and slows down in the late convergence process. However, the model in this paper has a steady downward trend, the oscillation amplitude is the smallest, and the convergence effect is the best, even with a small number of parameters, a stable training process can be achieved.

**Figure 13:**
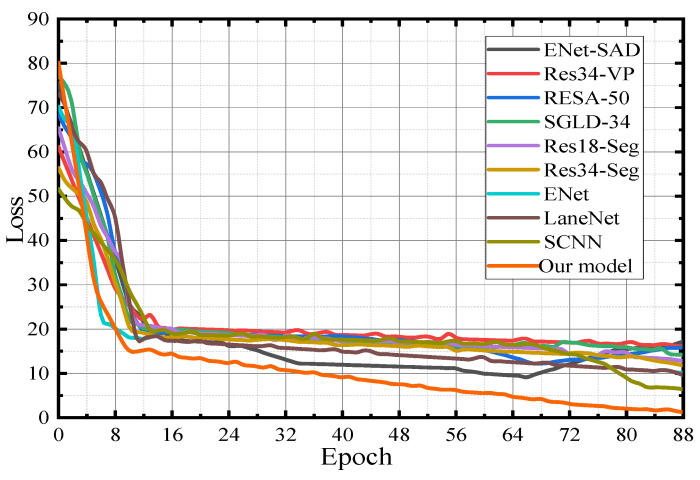
Model verification—loss function graph.

Figure 13 shows that in the training phase, the verification dataset is used to cross-validate each lane line detection model to generate a loss function verification curve. It can be seen from Figure 13 that ENet-SAD and RESA-50 present an overfitting phenomenon, according to the loss function curve which first decreases and then increases. At the same time, the other eight lane line detection models did not appear to be over-fitting during the training period, but during the training process, the loss function of the network such as Res34-VP has a certain degree of oscillation during the convergence process. Compared with the convergence effects of the remaining seven lane line detection models, the model in this paper has the best verification convergence.

**Figure 14:**
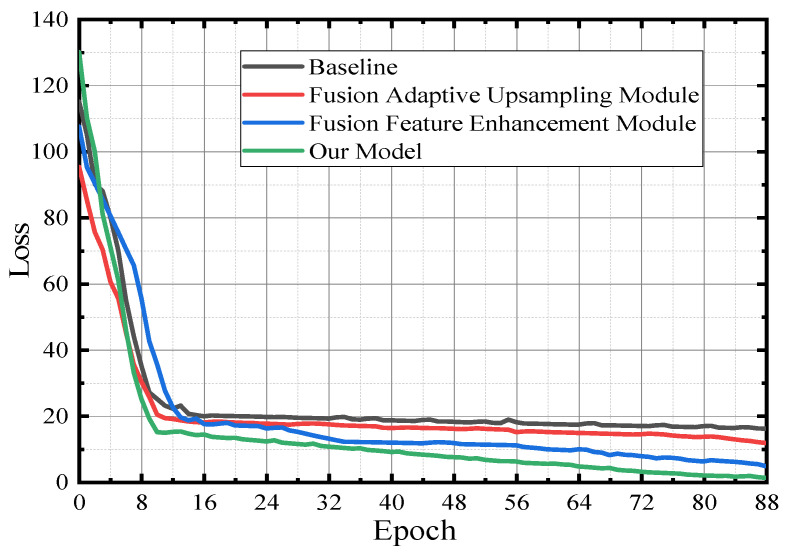
Model verification—loss function graph.

For the ablation experiments in Table 4, the performance before and after adding the adaptive upsampling module and feature enhancement module is comapared, to conduct a comparative analysis with the model in this paper. Figure 14 is the verification curve of the loss value during model training, and the experimental environment settings are the same as above. For the baseline, when the loss function converges to a specific stage, the convergence speed of the loss function decreases significantly due to the decline of the feature extraction ability of the model. After the convergence speed decreases, the baseline, adaptive upsampling module, and feature enhancement module, as well as the fusion feature enhancement module and adaptive upsampling module all show a state of convergence. However, the model in this paper has a stable downward trend, and the oscillation amplitude is the smallest, so convergence works best.

**Figure 15:**
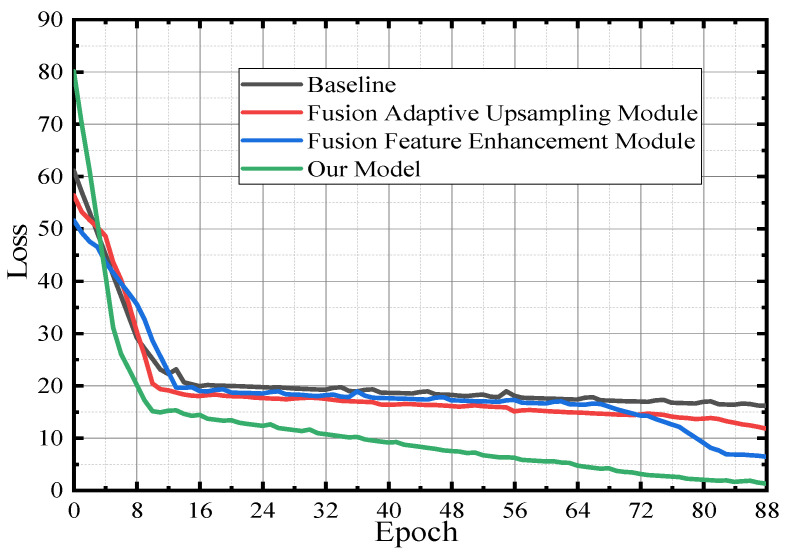
Model verification—loss function graph.

Figure 15 shows that in the training phase, the above four detection models are cross-validated using the verification data set to generate a loss function verification curve. It can be seen from Figure 15 that the model of the fusion feature enhancement module shows a significant increase in the convergence speed in the later stage, and the convergence effect outperforms the one fused with the baseline as well as networks fused with the adaptive upsampling module. However, the model in this paper has more advantages than the first three networks, so it has the best verification convergence.

### 3.5. Comparison of Lane Line Detection Effects

**Figure 16:**
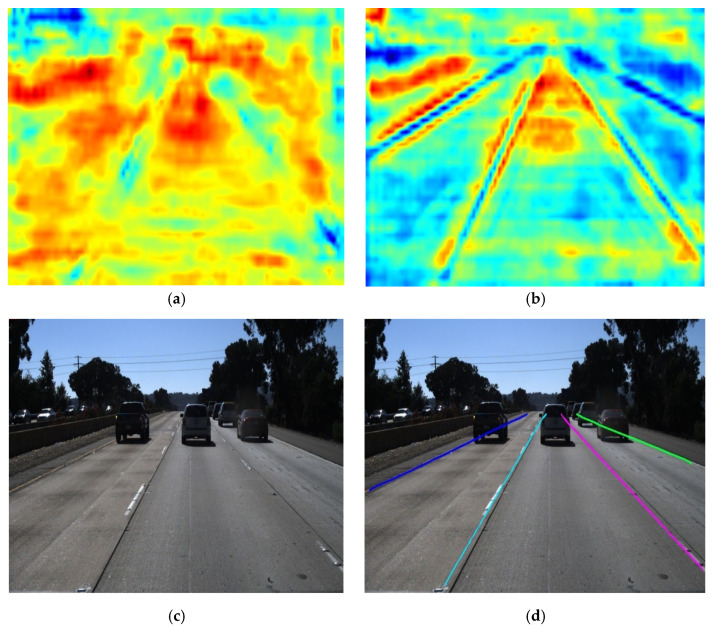
Visualization of the lane line detection process. (**a**) Before feature enhancement. (**b**) After feature enhancement. (**c**) Input image. (**d**) Detection result.

To verify the effectiveness of the feature enhancement module and the adaptive upsampling module, the feature maps before and after the feature enhancement module and the final detection results are visualized, as shown in Figure 16. Among them, Figure 16a is the feature map before the encoder processing, Figure 16b is the feature map after being processed by the feature enhancement module, and Figure 16c is the input image. Figure 16b shows that the features extracted by the encoder are relatively scattered local features, while the feature enhancement module can capture the complete features of the lane lines, and the perceived ability of the lane lines is significantly enhanced. Figure 16b is the mapping output by the adaptive upsampling module in the original image. It can be seen that the model can accurately detect the lane lines in the input image.

**Figure 17:**
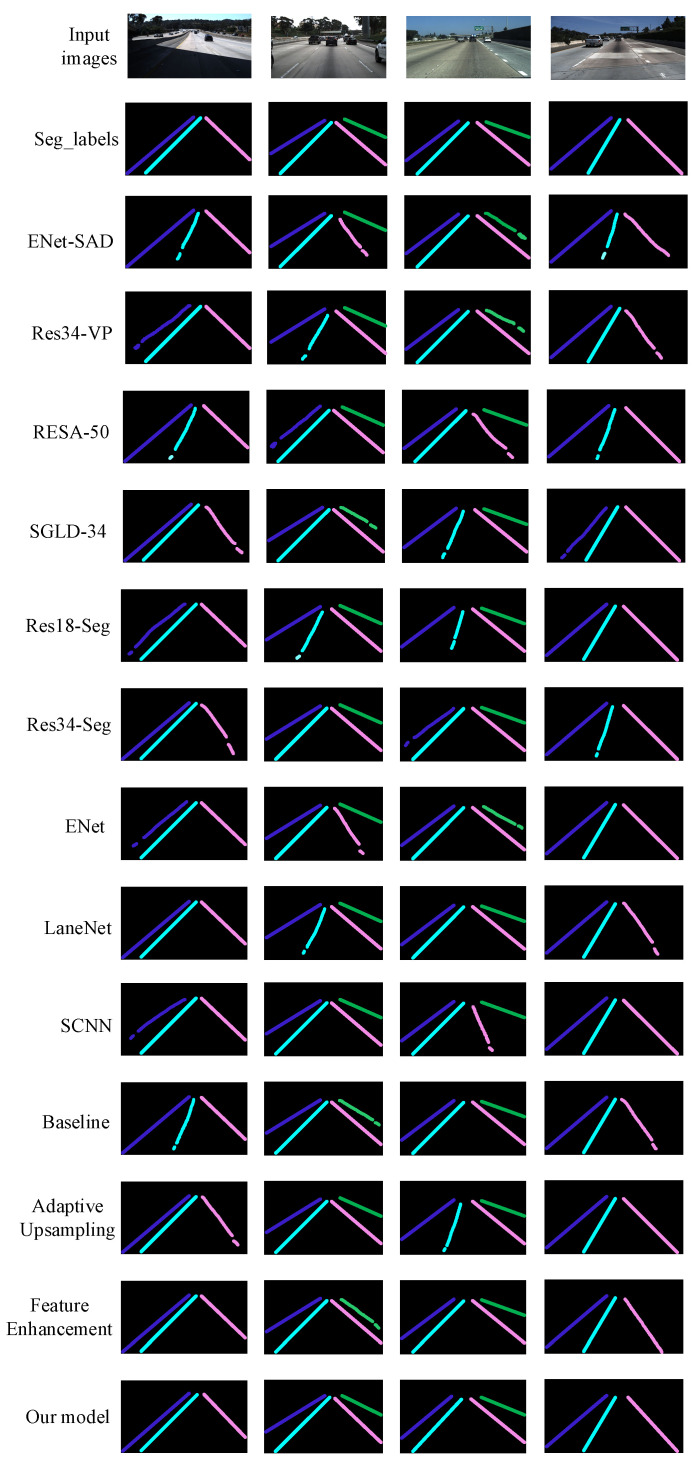
Instance Segmentation Analysis of Different Network Performance Based on TuSimple Dataset.

To verify the performance of the lane line detection model in this paper, the remaining nine lane line detection models in Table 3, and the first three in Table 4, different modules are fused. The four cases of road shadow, road blur, road occlusion, and road slender and sparse characteristics in the testset are selected for instance segmentation analysis, as shown in Figure 17 below. After inputting pictures and fixed labels, for the four lane line detection models of ENet-SAD, Res34-VP, RESA-50, and SGLD-34, the instances in the four scenarios are segmented, and the segmented solid lines have defect losses in the four scenarios. For the instance segmentation of Res18-Seg, Res34-Seg, and ENet three lane line detection models in four scenarios, the segmented solid lines have defect losses in three scenarios. For the instance segmentation of LaneNet and SCNN lane line detection models in four scenarios, the segmented solid lines have defect losses in two scenarios. For the instance segmentation of the three network models in the four scenes in the case of ablation experiments, the solid lines of the segmentation have defect losses in three scenes, two scenes, and one scene, respectively. Compared with the performance of the first 12 models, when the model in this paper performs instance segmentation on the four scenarios, there is no defect loss, and the detection effect reaches the best level. Therefore, on the whole, the lane detection model in this paper is superior to other lane detection models in terms of comprehensive performance.

### 3.6. Lane Line Detection and Mobile Terminal Deployment in Different Scenarios

**Figure 18:**
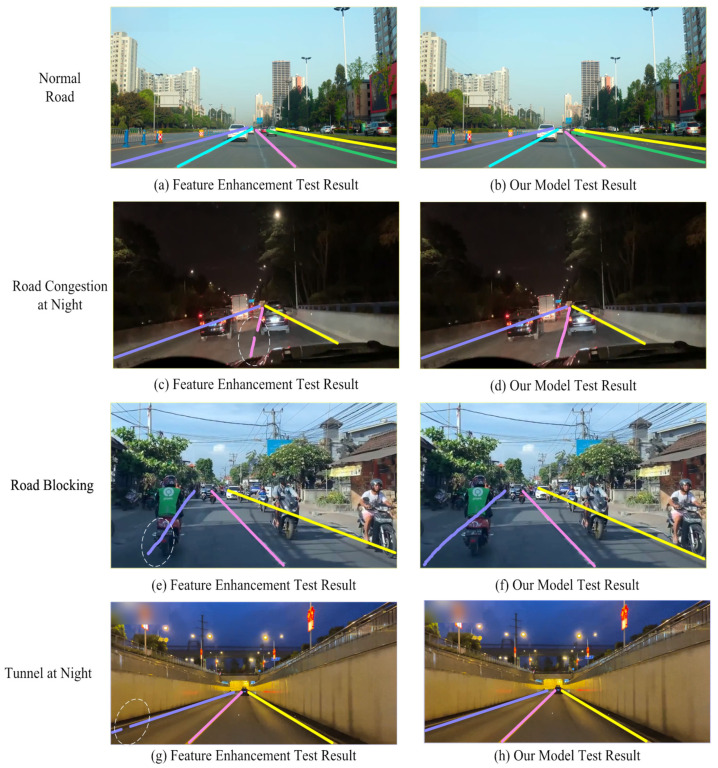
Lane line detection effect in different scenarios.

To further verify the effect of the lane line detection model in this paper, road video information is collected by the front car camera in different scenarios, such as normal roads, road congestion at night, road blocking, and night tunnels. At the same time, according to the results of instance segmentation under the TuSimple testset, the closest to the effect of the model in this paper is the network of the fusion feature enhancement module. Considering that there are many network models for comparison and reducing the repetition of experiments, the lane line detection model of the fusion feature enhancement module and the lane line detection model in this paper, for comparison and analysis in complex traffic scenarios, are shown in Figure 18.

It can be seen from Figure 18a,b that when driving on a normal road, both the lane line detection model of the fusion feature enhancement module and the lane line detection model in this paper can smoothly segment and accurately detect the lane line. The scenarios of road congestion at night, road blocking, and the tunnel at night can be seen in Figure 18c,e,g, showing that the lane line detection model with the fused feature enhancement module has a partial miss detection problem, which is marked with an elliptical dashed line. From Figure 18d,f,h, it can be seen that the lane line detection model in this paper can detect lane lines accurately.

**Figure 19:**
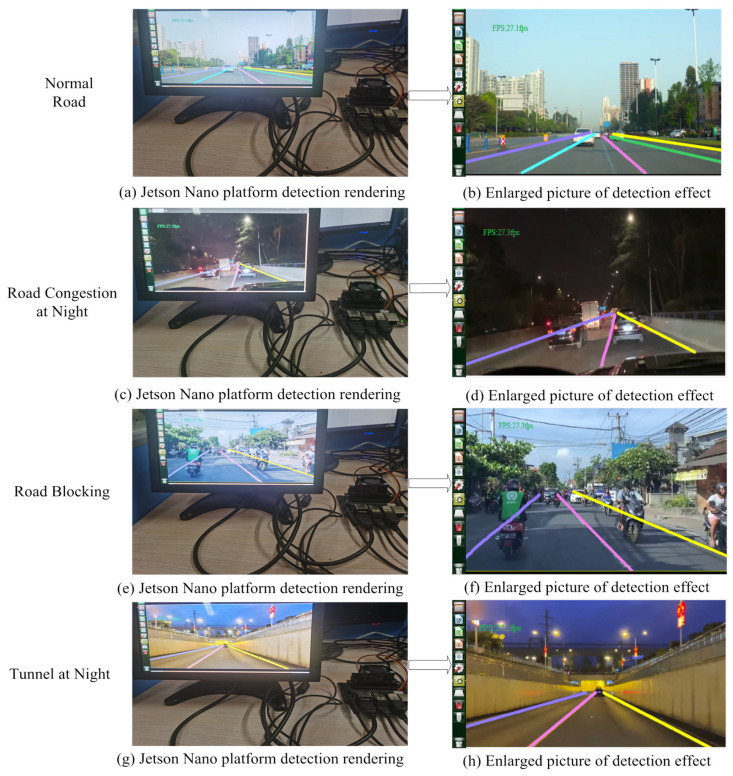
Mobile terminal detection rendering.

To further test the performance of the lane detection model in this paper, it is deployed on the mobile terminal for verification. It can be seen from Table 3 that the parameter quantity of the lane line model in this paper is 9.57 M, which is very low. At the same time, since RepVgg-A0 is a lightweight network, different branch structures are subtly fused during inference, thereby compressing the parameters of the model. Therefore, the lane line detection model can be directly deployed to the embedded platform Jetson Nano, and the TensorRT framework can be used for half-precision acceleration to make its detection speed meet the requirements of real-time detection. Based on the complex traffic scene in Figure 18, the lane line detection model in this paper is deployed to the embedded platform Jetson Nano, and the displayed results are shown in Figure 19 below.

Figure 19a,c,e,g show the deploying of the lane line detection model to the embedded platform Jetson Nano, and the performance of the model based on the above different scenarios can be tested. Due to the limited space shown in the picture on the left, its enlarged effect under the ubuntu18.0.4 system is shown in Figure 19b,d,f,h, in which it can be seen that under the premise of accurate detection of lane lines in complex scenarios, the real-time detection speed of the Jetson Nano platform has reached above 27 fps/s. Although there is still a certain gap with the real-time detection speed under the Windows system, it can meet the real-time detection speed requirements under the deployment of the mobile terminal. Therefore, the lane line detection model in this paper is deployed on the mobile terminal and performs well.

## 4. Conclusions

In this paper, aiming at the problems of low lane line detection accuracy and poor real-time detection speed of existing lane line detection algorithms in complex traffic scenes, a lane line detection algorithm based on instance segmentation is proposed. The design method of this paper mainly includes optimizing the RepVgg-A0 network structure to expand the receptive field of the network; a multi-size asymmetric shuffled convolution model is proposed to enhance extraction of sparse and slender lane lines ability; an adaptive upsampling model is proposed, which allows the network to select the weight of the two upsampling methods at each position; a lane line prediction branch is added to facilitate the output of lane line confidence; and the lane line detection algorithm is deployed to the embedding of the standard platform Jetson Nano, using the TensorRT framework for half-precision acceleration. The experimental results show that the lane line detection algorithm in this paper has an Acc value of 96.7% on the expanded TuSimple dataset and a real-time detection speed of 77.5 fps/s. The model is successfully deployed on the embedded platform Jetson Nano, and achieved a real-time detection speed of 27 fps/s, making it suitable for mobile terminal deployment. Therefore, the lane line detection algorithm in this paper is more suitable for current self-driving cars after being deployed on the mobile terminal, to improve the accuracy and safety of the automated driving perception part.

Due to the limitation of the experimental conditions, there is a gap between the real-time detection speed of the lane line algorithm deployed on the mobile terminal and the real-time detection speed under the Windows system. Therefore, the next step is to consider further compression of the model parameters, so that the real-time detection speed of the mobile terminal can be further improved without reducing the accuracy.

## Figures and Tables

**Table 1:** Overall structure of the encoder.

Stage	Output Size	Convolution Kernel	Step	Hole Rate	Number of Stacks	Output Channels
Input	368 × 640					3
Stage0	184 × 320	3 × 3	2	1	1	48
Stage1	92 × 160	3 × 3	2	1	2	48
Stage2	46 × 80	3 × 3	2	2	4	96
Stage3	46 × 80	3 × 3	1	2	14	192
Stage4	46 × 80	3 × 3	1	4	1	1280
Conv5	46 × 80	1 × 1	1	1	1	128

**Table 2:** Hyper parameter settings of model.

Name	Value
Batch size	8
Iterations	300
Initial learning rate	0.02
Optimizer	SGD
Optimizer decay factor	0.0001

**Table 3:** Parameter settings of model.

Net	Acc (%)	FP	FN	Params (M)	FLOPs (G)	FPS
ENet-SAD	90.13	0.0875	0.0810	11.12	56.33	75.1
Res34-VP	90.42	0.0891	0.0801	20.15	75.65	37.6
RESA-50	92.14	0.0876	0.0296	20.22	41.96	36.7
SGLD-34	92.18	0.0798	0.0589	13.56	45.12	59.3
Res18-Seg	92.69	0.0948	0.0822	12.03	42.63	79.3
Res34-Seg	92.84	0.0918	0.0796	22.14	79.88	34.2
ENet	93.02	0.0886	0.0734	0.95	2.20	72.6
LaneNet	96.38	0.0780	0.0224	20.66	111.31	35.8
SCNN	96.53	0.0617	0.0180	12.63	42.67	64.7
Our model	96.70	0.0359	0.0282	9.57	36.67	77.5

**Table 4:** Ablation experiment results.

Baseline	Adaptive Upsampling Module	Feature Enhancement Module	Acc/%
√			95.81
	√		96.01(+0.20)
		√	96.63(+0.82)
	√	√	96.70(+0.89)

## Data Availability

Not applicable.

## References

[1] Haris M., Hou J. (2020). Obstacle Detection and Safely Navigate the Autonomous Vehicle from Unexpected Obstacles on the Driving Lane. Sensors.

[2] Yang W., Zhang X., Lei Q. (2020). Lane Position Detection Based on Long Short-Term Memory (LSTM). Sensors.

[3] Mammeri A., Boukerche A., Tang Z. (2015). A real-time lane marking localization, tracking and communication system. Comput. Commun..

[4] Sotelo N., Rodríguez J., Magdalena L. (2004). A Color Vision-Based Lane Tracking System for Autonomous Driving on Unmarked Roads. Auton. Robot..

[5] Ozgunalp N., Dahnoun N. Lane detection based on improved feature map and efficient region of interest extraction. Proceedings of the 2015 IEEE Global Conference Signal and Information Process (GlobalSIP, IEEE 2015).

[6] Chi F.H., Huo Y.H. Forward vehicle detection system based on lane-marking tracking with fuzzy adjustable vanishing point mechanism. Proceedings of the 2012 IEEE International Conference on Fuzzy Systems.

[7] Lin H.Y., Dai J.M., Wu L.T., Chen L.Q. (2020). A Vision-Based Driver Assistance System with Forward Collision and Overtaking Detection. Sensors.

[8] Li K., Shao J., Guo D. (2019). A Multi-Feature Search Window Method for Road Boundary Detection Based on LIDAR Data. Sensors.

[9] Cao Y., Chen Y., Khosla D. (2014). Spiking deep convolutional neural networks for energy-efficient object Recognition. Int. J. Comput. Vis..

[10] Zhang X., Yang W., Tang X., Wang Y. (2019). Lateral distance detection model based on convolutional neural network. IET Intell. Transp. Syst..

[11] Kim J., Kim J., Jang G.-J., Lee M. (2017). Fast learning method for convolutional neural networks using extreme learning machine and its application to lane detection. Neural Netw..

[12] Aly M. Real time detection of lane markers in urban streets. Proceedings of the IEEE Intelligent Vehicles Symposium.

[13] Kim J., Park C. End-To-End Ego Lane Estimation Based on Sequential Transfer Learning for Self-Driving Cars. Proceedings of the 2017 IEEE Conference on Computer Vision and Pattern Recognition Workshops (CVPRW, 2017).

[14] Neven D., de Brabandere B., Georgoulis S.M. Towards End-to-End Lane Detection: An Instance Segmentation Approach. Proceedings of the 2018 IEEE Intelligent Vehicles Symposium(IV).

[15] Ren S., He K., Girshick R., Sun J. (2017). Faster R-CNN: Towards Real-Time Object Detection with Region Proposal Networks. IEEE Trans. Pattern Anal. Mach. Intell..

[16] He K., Zhang X., Ren S., Sun J. (2014). Spatial Pyramid Pooling in Deep Convolutional Networks for Visual Recognition. IEEE Trans. Pattern Anal. Mach. Intell..

[17] Haris M., Jin H., Xiao W. (2022). Lane line detection and departure estimation in a complex environment by using an asymmetric kernel convolution algorithm. Vis. Comput..

[18] Liu R., Yuan Z., Liu T. End-to-end lane shape prediction with transformers. Proceedings of the IEEE Winter Conference on Applications of Computer Vision.

[19] Chao M., Dean L., He H. (2021). Lane Line Detection Based on Improved Semantic Segmentation. Sens. Mater..

[20] Ma N., Zhang X., Zheng H.T. Shufflenet v2: Practical guidelines for efficient CNN architecture design. Proceedings of the European Conference on Computer Vision (ECCV).

[21] Ding X., Zhang X., Ma N., Han J., Ding G., Sun J. Repvgg: Making vgg-style convnets great again. Proceedings of the IEEE/CVF Conference on Computer Vision and Pattern Recognition.

[22] Qiu S., Xu X., Cai B. FReLU: Flexible rectified linear units for improving convolutional neural networks. Proceedings of the 2018 24th International Conference on Pattern Recognition (ICPR).

[23] Szegedy C., Vanhoucke V., Ioffe S. Rethinking the inception architecture for computer vision. Proceedings of the IEEE Conference on Computer Vision and Pattern Recognition.

[24] Zheng T., Fang H., Zhang Y. Resa: Recurrent feature-shift aggregator for lane detection. Proceedings of the AAAI Conference on Artificial Intelligence.

[25] Romera E., Alvarez J.M., Bergasa L. (2017). Erfnet: Efficient residual factorized convnet for real-time semantic segmentation. IEEE Trans. Intell. Transp. Syst..

[26] Wang P., Chen P., Yuan Y. Understanding convolution for semantic segmentation. Proceedings of the 2018 IEEE Winter Conference on Applications of Computer Vision (WACV), IEEE.

[27] Chen L.C., Papandreou G., Kokkinos I. (2017). Deeplab: Semantic image segmentation with deep convolutional nets, atrous convolution, and fully connected crfs. IEEE Trans. Pattern Anal. Mach. Intell..

[28] Paszke A., Chaurasia A., Kim S. (2016). Enet: A deep neural network architecture for real-time semantic segmentation. arXiv.

[29] Lu P., Xu S., Peng H. (2021). Graph-Embedded Lane Detection. IEEE Trans. Image Process..

[30] Pan X., Shi J., Luo P. Spatial as deep: Spatial cnn for traffic scene understanding. Proceedings of the AAAI Conference on Artificial Intelligence.

[31] Hou Y., Ma Z., Liu C. Learning lightweight lane detection CNNS by self attention distillation. Proceedings of the IEEE International Conference on Computer Vision.

[32] Wang B., Wang Z., Zhang Y. Polynomial regression network for variable-number lane detection. Proceedings of the European Conference on Computer Vision.

[33] Su J., Chen C., Zhang K. (2021). Structure guided lane detection. arXiv.

[34] Liu Y.B., Zeng M., Meng Q.H. (2020). Heatmap-based vanishing point boosts lane detection. arXiv.

